# Supplementary material for: Sociodemographic Correlates of Obesity among Spanish Schoolchildren: A Cross-Sectional Study
Source: Children (Basel). 2020 Oct 28;7(11):201. doi: 10.3390/children7110201 (PMC7692605; doi:10.3390/children7110201)
Supplement: Supplementary file 1 [file children-07-00201-s001.pdf]

Supplementary data

Table S1. Prevalence of having excess weight, abdominal obesity, high trunk fat mass, and high adiposity according to the different sociodemographic factors.

Variables	*Excess weight (BMI)* [a]	*p*	*Abdominal obesity (WHtR)*	*p*	*High trunk mass fat (WC)*	*p*	*High adiposity (%BF)*	*p*
**Age group**								
6-9	120 (52.2)	0.092	35 (15.2)	0.664	47 (20.4)	0.920	85 (37.0)	<0.001
10-13	74 (52.9)		19 (13.6)		28 (20.0)		83 (59.3)	
**Sex**								
Boys	114 (55.9)	0.086	27 (13.2)	0.412	43 (21.1)	0.668	88 (43.1)	0.331
Girls	80 (48.2)		27 (16.3)		32 (19.3)		80 (48.2)	
**Type of schooling**								
Public	141 (57.1)	0.008	45 (18.2)	0.005	58 (23.5)	0.029	120 (48.6)	0.082
Private	53 (43.1)		9 (7.3)		17 (13.8)		48 (39.0)	
**PIR**								
High PIR	180 (65.3)	0.042	51 (19.4)	0.330	70 (26.4)	0.250	151 (45.0)	0.930
Low PIR	14 (49.3)		3 (13.4)		5 (18.8)		17 (46.6)	
**Area of residence**								
Urban	152 (36.8)	0.187	41 (7.9)	0.539	54 (13.2)	0.082	127 (44.7)	0.446
Rural	42 (53.9)		13 (15.4)		21 (21.1)		41 (45.5)	
**Immigrant status**								
Immigrant	47 (53.9)	0.015	14 (14.5)	0.194	19 (19.1)	0.150	42 (58.3)	0.014
Native	147 (47.7)		40 (14.8)		56 (23.9)		126 (42.3)	

Data expressed as number (percentage). BMI: Body mass index; BF: Body fat; PIR: per capita income; WHO: World Health Organization; WC: Waist circumference; WHtR: Waist-to-height ratio. Adjusted by age group, sex, type of schooling, PIR, area of residence and immigrant status. [a] Excess weight determined by WHO criteria [6].

Table S2. Association of having excess weight, abdominal obesity, high trunk fat mass and high adiposity according to different sociodemographic factors.

Variables	*Excess weight (BMI)* [a]	*Abdominal obesity (WHtR)*	*High trunk mass fat (WC)*	*High adiposity (%BF)*
**Age group**				
6-9	1.00 (0.65-1.55)	0.87 (0.47-1.62)	0.95 (0.55-1.62)	0.38** (0.24-0.59)
10-13 (Ref.)	1	1	1	1
**Sex**				
Boys	1.35 (0.88-2.05)	0.87 (0.47-1.62)	1.13 (0.67-1.91)	0.72 (0.47-1.11)
Girls (Ref.)	1	1	1	1
**Type of schooling**				
Public	1.96* (1.19-3.20)	0.76 (0.42-1.38)	1.73 (0.90-3.31)	1.35 (0.81-2.25)
Private (Ref.)	1	1	1	1
**PIR**				
High PIR	2.29 (0.96-5.48)	3.12* (1.40-6.94)	1.48 (0.79-2.79)	1.23 (0.52-2.94)
Low PIR (Ref.)	1	1	1	1
**Area of residence**				
Urban	1.13 (0.59-2.19)	2.90 (0.74-11.44)	0.58 (0.28-1.19)	0.99 (0.51-1.94)
Rural (Ref.)	1	1	1	1
**Immigrant status**				
Immigrant	1.65 (0.94-2.88)	1.26 (0.62-2.55)	1.90* (1.10-3.30)	1.90* (1.09-3.32)
Native (Ref.)	1	1	1	1

Data expressed as odds ratio (confident intervals 95%). BMI: Body mass index; BF: Body fat; PIR: per capita income; WHO: World Health Organization; WC: Waist circumference; WHtR: Waist-to-height ratio. Adjusted by age group, sex, type of schooling, PIR, area of residence and immigrant status. [a] Excess weight determined by WHO criteria [6]. * $p < 0.050$; ** $p < 0.001$.
